# Full-Spectrum CARS Microscopy of Cells and Tissues with Ultrashort White-Light Continuum Pulses

**DOI:** 10.1021/acs.jpcb.3c01443

**Published:** 2023-05-17

**Authors:** Federico Vernuccio, Renzo Vanna, Chiara Ceconello, Arianna Bresci, Francesco Manetti, Salvatore Sorrentino, Silvia Ghislanzoni, Flavia Lambertucci, Omar Motiño, Isabelle Martins, Guido Kroemer, Italia Bongarzone, Giulio Cerullo, Dario Polli

**Affiliations:** † Department of Physics, Politecnico di Milano, Piazza Leonardo da Vinci 32, 20133 Milan, Italy; ‡ CNR-Institute for Photonics and Nanotechnologies (IFN-CNR), Piazza Leonardo Da Vinci 32, 20133 Milan, Italy; § MALDI-imaging Lab, Department of Advanced Diagnostics, Fondazione IRCCS Istituto Nazionale dei Tumori, Via G. Amadeo 42, 20133, Milan, Italy; ∥ Centre de Recherche des Cordeliers, Equipe Labellisée par la Ligue Contre le Cancer, Inserm U1138, Université Paris Cité, Sorbonne Université, 75006 Paris, France; ⊥ Metabolomics and Cell Biology Platforms, Gustave Roussy, 94805 Villejuif, France; # Institut du Cancer Paris CARPEM, Department of Biology, Hôpital Européen Georges Pompidou HP, 75015 Paris, France

## Abstract

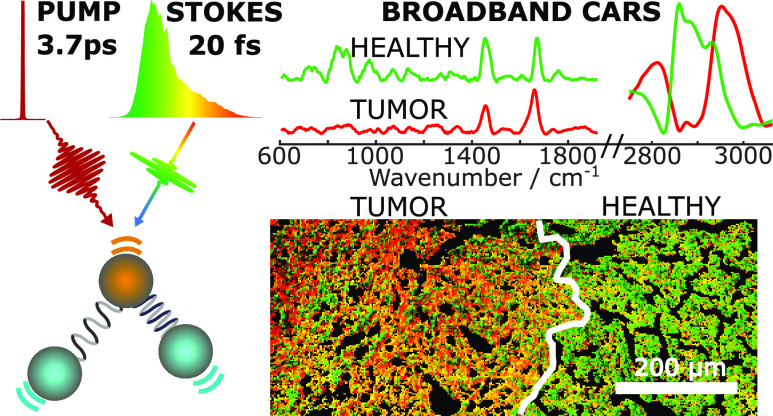

Coherent anti-Stokes Raman scattering (CARS) microscopy is an emerging nonlinear vibrational imaging technique that delivers label-free chemical maps of cells and tissues. In narrowband CARS, two spatiotemporally superimposed picosecond pulses, pump and Stokes, illuminate the sample to interrogate a single vibrational mode. Broadband CARS (BCARS) combines narrowband pump pulses with broadband Stokes pulses to record broad vibrational spectra. Despite recent technological advancements, BCARS microscopes still struggle to image biological samples over the entire Raman-active region (400–3100 cm^–1^). Here, we demonstrate a robust BCARS platform that answers this need. Our system is based on a femtosecond ytterbium laser at a 1035 nm wavelength and a 2 MHz repetition rate, which delivers high-energy pulses used to produce broadband Stokes pulses by white-light continuum generation in a bulk YAG crystal. Combining such pulses, pre-compressed to sub-20 fs duration, with narrowband pump pulses, we generate a CARS signal with a high (<9 cm^–1^) spectral resolution in the whole Raman-active window, exploiting both the two-color and three-color excitation mechanisms. Aided by an innovative post-processing pipeline, our microscope allows us to perform high-speed (≈1 ms pixel dwell time) imaging over a large field of view, identifying the main chemical compounds in cancer cells and discriminating tumorous from healthy regions in liver slices of mouse models, paving the way for applications in histopathological settings.

## Introduction

Vibrational microscopy is a powerful investigation tool in life sciences, as it delivers vibrational maps of unstained tissues and cells, providing high chemical specificity in a label-free and nondestructive manner.^1−4^ Among vibrational microscopy techniques, coherent anti-Stokes Raman scattering (CARS)^5−9^ has gained prominence, since it enables high-speed imaging thanks to the nonlinear nature of the optical processes occurring at the sample under tight-focusing conditions. With respect to spontaneous Raman (SR), in which a monochromatic excitation beam interacts with thermal vibrations, in CARS two synchronized, spatially overlapped and frequency detuned pulses, the pump (at frequency ω_p_) and the Stokes (at frequency ω_S_), generate a vibrational coherence at the frequency Ω = ω_p_ – ω_S_. This coherence is read by a further interaction with the pump beam, resulting in the emission of a coherent beam at the anti-Stokes frequency ω_aS_ = ω_p_ + Ω, which is blue-shifted with respect to the pump and Stokes frequencies and thus, unlike SR, is not spectrally overlapped with the autofluorescence of biological samples.

In the simplest, single-frequency approach to CARS, pump and Stokes are narrowband and only one vibrational mode at the time is interrogated. Thanks to its nonlinear nature, CARS generates signals only in the small focal volume, thus providing 3D sectioning capability without the use of any confocal configuration and enhances the signal by orders of magnitude with respect to SR, allowing for fast scanning rates. However, the CARS signal sits on top of a chemically unspecific radiation, known as nonresonant background (NRB), which derives from a four-wave-mixing process between pump and Stokes pulses mediated by the nonresonant third-order optical nonlinearity of the sample. The NRB distorts and shifts the resonant vibrational peaks of the investigated specimen and, in single-frequency CARS, is difficult to distinguish from the resonant signal. On the other hand, it acts as a self-heterodyned amplifier of the resonant signal,^10^ which can be recovered if the complete CARS spectrum is available.

Broadband CARS (BCARS)^11^ may be implemented either by coupling narrowband pump pulses with tunable narrowband Stokes pulses (hyperspectral CARS),^12,13^ whose frequency detuning sequentially matches the Raman modes of the samples, or by combining narrowband pump pulses with broadband Stokes pulses (multiplex CARS),^10,14−21^ thus interrogating multiple vibrational modes in parallel. Nowadays, the state of the art for multiplex BCARS microscopy covering the entire vibrational spectrum in biological samples mainly refers to frequency-domain CARS. In this respect, we mention the pioneering work by Camp et al.^10^ Their experimental setup was based on a mode-locked 40 MHz Er:fiber laser system delivering 3.4 ps pump pulses at 780 nm and synchronized near-infrared Stokes pulses with a 16 fs duration at the sample, obtained via supercontinuum generation in a highly nonlinear fiber. This configuration enables mapping the entire Raman window (500–3500 cm^–1^) of biological tissues with a 3.5 ms pixel dwell time. Recently, Kano et al.^15,22^ reported a high-speed multiplex CARS microscope covering from 600 to 3600 cm^–1^ with unprecedented speed (≈1.8 ms) when imaging cells. They employed a Q-switched Nd:YVO_4_ microchip laser oscillator generating sub-100 ps laser pulses at a 0.82 MHz repetition rate, which produced a supercontinuum in a photonic crystal fiber (PCF).

BCARS spectra can also be recorded using time-domain approaches, such as Fourier-transform CARS (FT-CARS).^14,23−25^ In FT-CARS, the vibrational coherence is generated via impulsive stimulated Raman scattering^26^ (ISRS) using an ultrashort pump pulse and probed by a replica of the pulse at various pump-probe delays. FT-CARS has the advantage of suppressing the NRB contribution by removing the signals around time zero, where the nonresonant electronic responses can be found. While frequency-domain BCARS typically employs slow (limited to ≈1 ms readout time) CCD sensors, FT-CARS spectroscopy enables high acquisition speed, up to ≈24,000 spectra/s in solvents,^14^ as it records the signal using a fast single-channel detector. However, to the best of our knowledge, only a few examples of FT-CARS microscopy detecting the fingerprint region of biological samples have been reported in the literature. As an example, Kinegawa et al.^27^ demonstrated BCARS at an unprecedentedly low pixel dwell time, down to 42 μs, but at a spectral resolution of only 37 cm^–1^, much worse than the one usually required in the fingerprint region (<10 cm^–1^), featuring very narrow and congested lineshapes.^28^

We recently introduced a new approach to high-speed multiplex CARS microscopy based on a 2 MHz repetition rate-amplified ytterbium laser system, producing the broadband Stokes pulses through white-light continuum (WLC) generation in bulk media.^29^ Compared to previous multiplex CARS systems operating with a higher repetition rate (≈40–80 MHz) and nJ energy pulses, our platform employs much higher pulse energies (≈ 2 μJ), thus enabling us to replace the PCFs used for the broadband Stokes beam generation with a bulk yttrium aluminum garnet (YAG) crystal. This solution leads to a setup that is more robust, compact, and alignment insensitive. Furthermore, the generated WLC shows long-term stability and does not suffer from wavelength-dependent intensity fluctuations that may affect the shape of the CARS spectrum. The use of high pulse energies also results in stronger CARS signals, improving the signal quality and acquisition speed of the setup.^30^

Our first demonstration of multiplex CARS with supercontinuum generation in bulk media, using frequency-chirped Stokes pulses, worked according to a so-called two-color scheme, exploiting the inter-pulse interaction between the pump and Stokes beams, whose frequency differences match the vibrational modes of the sample.^11^ This configuration provided good performance in retrieving Raman spectra covering the whole fingerprint region of test chemical analytes, such as solvents and polymer beads, and of mouse bone samples and demonstrated unprecedented sensitivity (≈ 14 mmol/L) and state-of-the-art speed (<1 ms/pixel) for multiplex CARS. However, no spectra of cells and tissues were presented and the system did not allow us to cover simultaneously the fingerprint and the CH stretching region.

Here, we present a novel and improved configuration for multiplex CARS microscopy with WLC generated in a bulk medium, which can image cells and tissues recording vibrational spectra spanning from 500 to 3100 cm^–1^. The setup combines narrowband (<9 cm^–1^ FWHM spectral resolution) pump pulses at 1035 nm with pre-compressed broadband (≈1200–1600 nm) Stokes pulses, featuring a nearly transform-limited (TL) <20 fs pulse duration at the focal plane of the first microscope objective. The use of ultrashort Stokes pulses with a finely tuned duration and an adjustable spectrum enables us to cover the lower frequencies of the vibrational spectrum (from 500 to 1400 cm^–1^) through the so-called three-color CARS mechanism,^31^ using the ultrashort Stokes pulse for impulsive excitation of the vibrational modes, and the narrowband pump beam as a probe. The higher-frequency portion of the Raman spectrum, from 1400 to 3100 cm^–1^, is covered through the classical two-color mechanism via inter-pulse excitation between the narrowband pump and the broadband Stokes beam. We deliver high-quality multiplex CARS images on HepG2 cells and thin slices of the liver tissue of mouse models, with a pixel dwell time down to 1 ms. These results demonstrate that our platform is able to deliver chemical maps of heterogeneous biological samples over a large field of view (≈800 × 800 μm^2^) with great potential impact in histopathological applications.

## Materials and Methods

### Data Acquisition and Post-Processing

The BCARS images are acquired by raster scanning the sample, synchronizing the stage movement with the CCD acquisition using an external trigger. To localize the region of the sample to be imaged, we equipped the microscope with a Köhler illumination scheme. The system acquires hyperspectral data, i.e., three-dimensional hypercubes, with 1340 spectral points in the third dimension.

For all the measurements shown in this work, we adopted the same experimental method that may be divided into several steps. First, we acquire 100 CARS spectra of toluene, 100 spectra focusing on a quartz coverslip that we consider as NRB spectra, and 100 dark spectra with 1 ms exposure time. We average the three sets of spectral data, and we use them to calibrate the wavenumber axis of our system. We subtract the averaged dark spectrum from the CARS data and then remove the NRB through the time-domain Kramers–Kronig algorithm.^32^ Subsequently, we compare the retrieved resonant Raman spectrum of toluene with the SR spectrum acquired averaging five times 5 s acquisitions with a home-built SR experimental setup described in our previous works.^29,33^

To extract the relevant biochemical information, getting rid of the spurious noise and of the unspecific NRB, we process the raw data using a pipeline consisting of three steps: data denoising, removal of the NRB, and spectral unmixing. A denoiser can be based on singular-value decomposition (SVD),^34^ spectral total variation,^35^ or machine-learning models^29,36−38^ trained with pairs of noisy inputs and ideal outputs to find the best parameters that minimize the error between the predicted output and the ideal one. In this work, we employed SVD.^10,39^ The three-dimensional BCARS dataset (*m* × *n* × *s*, where *m* and *n* are the numbers of spatial pixels along the *x* and *y* directions, respectively, and *s* is the number of spectral points) is unfolded into a two-dimensional matrix *M*, whose rows represent the spectral axis with *s* points and whose columns represent the spatial pixels (*p* = *m* × *n*). The SVD algorithm factorizes this matrix into three components:
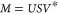
1where *U* is a two-dimensional (*s* × *s*) matrix, which contains the spectral bases (orthonormal eigenvectors), *S* is a diagonal (*s* × *s*) matrix containing the “singular values” (eigenvalues) ordered from the larger to the smaller, *V* (*p* × *s*) describes the spatial distribution of the bases in *U*, and “*” is the conjugate transpose. To remove the spurious noise, we defined an arbitrary cutoff, setting to zero all the eigenvalues below the threshold values. The modified *Ŝ* matrix is then used to reconstruct the denoised matrix *M̂* as follows: *M̂* = UŜV*. It is worth noticing that the SVD works properly only if the noise is additive and follows a Gaussian distribution.^34^ However, BCARS data typically exhibit a Poisson–Gaussian noise. Therefore, before performing the SVD, we apply an Anscombe transformation^37^ that acts as a variance stabilizer, whitening the noise of the data. After the reconstruction of matrix *M̂*, we apply the inverse Anscombe transformation to restore the BCARS spectra with mixed noise.

Our second post-processing step consists in the removal of the NRB, which can be achieved using either numerical algorithms^40,41^ or machine-learning based models.^42−44^ For the data shown in this paper, we applied a time-domain Kramers–Kronig algorithm.^32,40^ Eventually, once spectra comparable to those measured with SR are obtained and all the relevant peaks are identified, the spectral unmixing algorithm can be employed. This can be implemented either reducing the dimensionality of the data by defining a new basis of the main representative spectra and the relative abundances in each pixel, through multivariate curve resolution analysis^45^ or the N-FINDR algorithm,^46^ or grouping the pixels in a certain number of clusters through *k*-means cluster analysis or hierarchical cluster analysis.^47^ We distinguished the main constituents in heterogeneous biological samples using the N-FINDR algorithm.^2,3^ This method allows us to reduce the dimensionality of the hyperspectral data by finding the combination of spectra, called endmembers, which best represents the whole image. The algorithm is based on the maximization of a quantity, called the volume, whose expression is
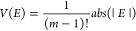
2where *m* is the number of endmembers and *E* is the augmented endmember matrix. Defining the *i*th endmember as *e_i_*, *E* is
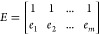
3

The algorithm starts with a random set of vectors. To find the endmembers, every pixel in the image must be evaluated as to its likelihood of being a pure or nearly pure pixel. This is done by calculating the volume with each pixel in place of each endmember. If the replacement results in an increase in volume, the pixel replaces the endmember. This procedure is repeated until there are no more replacements of endmembers. Once the endmembers are found, the spectra constitute the new basis onto which to project the initial image, using a nonnegatively constrained least-squares algorithm. This last step finds the abundances or concentration maps of the reduced dataset. The number of components (i.e., the chemical species) in the N-FINDR algorithm is arbitrarily selected, and its choice depends on the heterogeneity of the investigated sample. The main goal when using the spectral unmixing algorithm is to group pixels sharing similar chemical information. When the selected number of components is higher than the effective number of distinct chemical species on the sample, the algorithm starts grouping the pixels in classes not according to different spectral features but according to different signal intensities and, in some cases, noise due to environmental or laser fluctuations. For the data shown in this manuscript, we chose the number of components depending on how many different chemical species we can derive out of our BCARS images. We denoised the data and removed the NRB contribution using MATLAB, while the analysis of the processed hyperspectral data to produce false-color images was performed using a web-based tool developed internally, called “RamApp”,^48^ that integrates different algorithms into an interactive graphical user interface.

### Sample Preparation

#### HepG2 Cells

Cells from the hepatocellular carcinoma cell line HepG2 were purchased from the American Type Culture Collection (ATCC; Manassas, Virginia, USA; ATCC number: HB-8065) and maintained in Dulbecco’s modified Eagle’s medium (DMEM; Gibco, Italy) supplemented with 10% fetal bovine serum (FBS; Gibco) at 37 °C and 5% CO_2_. For BCARS microscopy, cells were plated on 170 μm-thick quartz coverslips at the density of 320,000 cells/mL and treated with 100 μM deferoxamine (DFO; Merck, Darmstadt, Germania). 72 h after DFO treatment, the cells were fixed in 4% paraformaldehyde (PFA, Merck) for 10 min and stored at −4 °C. For the measurements, we adopted a sandwich configuration: a drop of phosphate-buffered saline (PBS, Gibco) buffer was added to the cells, and a second 170 μm-thick quartz coverslip was positioned on the top and then fixed with enamel glue.

#### Mouse Models

2–3-month-old male C57BL/6 J mice were housed under temperature-controlled conditions and provided with food and water ad libitum. Experimentations were conducted under the FELASA guidelines and approved by the Ethics committee of INSERM and Centre de Recherche des Cordeliers (project numbers 25000, 31411, and 31313).

#### Control and NASH Murine Models

To induce the nonalcoholic steatohepatitis (NASH) model, mice were fed with a methionine- and choline-deficient (MCD) diet, which produces the most severe NASH phenotype in the quickest timeframe. This is a diet composed of high sucrose (40%) and fat (10%) content but without methionine and choline. Choline deficiency induces an accumulation of lipids in the liver, and methionine deficiency causes oxidative stress and contributes to disease progression.^49^ In detail, 8-week-old male C57BL/6 J mice were fed either with regular chow diet (RCD), for control murine models, or with MCD diet (MCD; AIN-76 Safe diet, Essingen, Germany), for NASH murine models, and after 4 weeks, all mice were euthanized by cervical dislocation.

#### Tumor-Bearing Liver

The orthotopic model used to study liver cancer ex vivo was prepared by implanting hepatocellular carcinoma (HCC) tumor cells into the liver of mice by direct intrahepatic injection to generate HCC. In this model, tumors occur in the natural liver microenvironment and mimic the metastatic behavior of HCC. 7-week-old C57BL/6 J female mice were anesthetized with 2% isoflurane, and their abdomen was opened to expose the liver. Then, 30 × 10^4^ Hep55.1C-Luc c cells were injected orthotopically into the left-lateral hepatic lobe. Sutures were used to close the peritoneum and wound clips to close the skin. Tumor growth was monitored during the following weeks, and finally, mice were euthanized 6 weeks after implantation by cervical dislocation.

#### Tissue Sample Preparation before Standard Assessment and CARS Experiments

In the murine models (i.e., control, NASH, and liver tumor models), liver tissues were harvested, embedded in optical cutting temperature (OCT) compound, and simultaneously frozen in liquid nitrogen. Serial 10 μm-thick sections of frozen tissue were cut with a cryostat (Leica, France) and alternately deposited on glass slides, for hematoxylin and eosin (H&E) standard assessment, or 170 μm-thick quartz coverslips, for CARS experiments. Before H&E staining, tissue slices were deparaffinized in xylene and rehydrated in a degressive series of ethanol solutions. The samples were incubated in Mayer’s hemalum (VWR, 720-0330) solution for 4 min 30 s and then rinsed in distilled water. The slides were then stained with eosin solution (MM France, 6766010) for 2 min 30 s and rinsed in water and 100% ethanol. Subsequently, the specimens were incubated in the saffron solution (VWR, 720-0184) for 3 min 30 s and rinsed in 100% ethanol. Slides were dehydrated with three successive baths of 100% ethanol and xylene. The slides were mounted with cover slides with Pertex (a permanent mounting medium). For CARS experiments, tissue slices adjacent to the stained one were analyzed without further processing or staining but only covering the slice with another 170 μm-thick quartz coverslip.

## Results and Discussion

### BCARS Microscope

**Figure 1:**
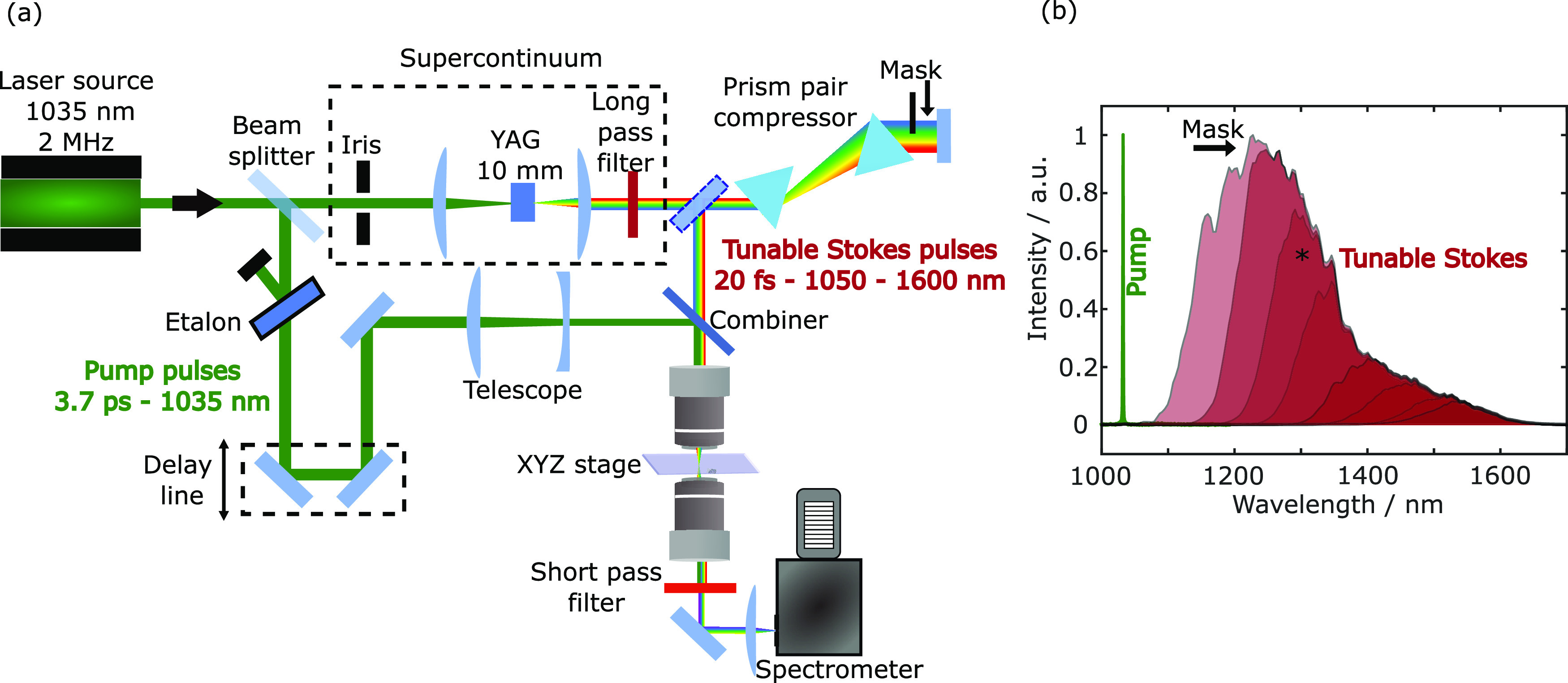
(a) Experimental scheme of the multiplex CARS microscope (see text for details). (b) Spectrum of the pump (green) and Stokes (red) beam measured varying the insertion of the mask after the second prism of the compressor. The * marks the spectrum of the broadband Stokes beam selected for the subsequent measurements.

**Figure 2:**
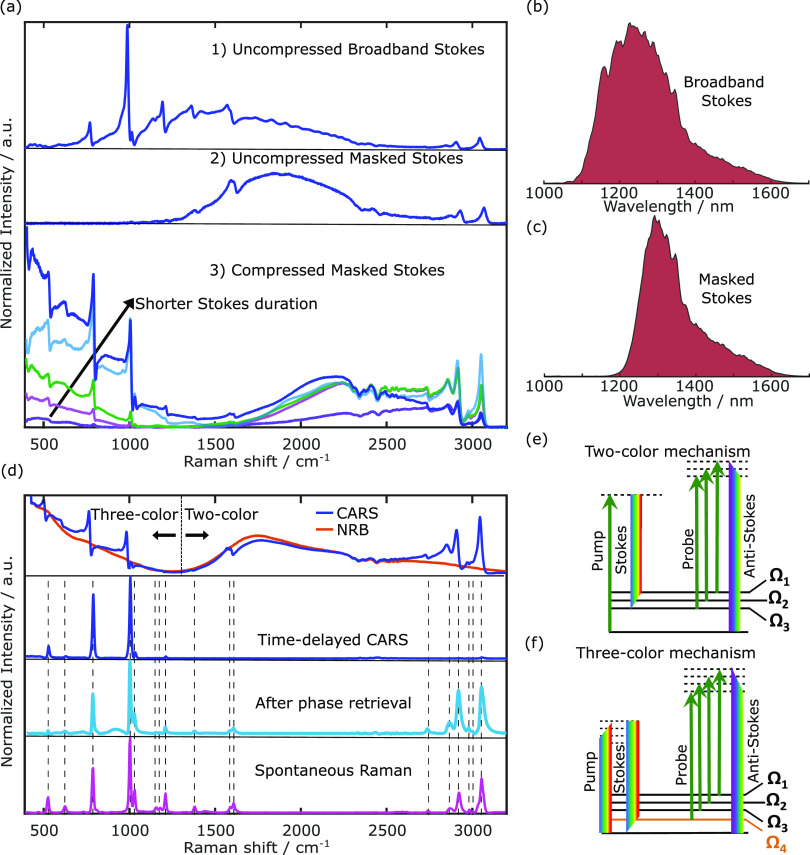
(a) CARS spectrum of toluene sandwiched between two quartz coverslips and acquired with 1 ms exposure time in three different setup configurations: using the whole bandwidth of the Stokes beam (b) without compressing it (two-color CARS only); using a mask that reduces the frequency span of the Stokes spectrum (c) without compressing it (two-color CARS only); using the Stokes in (c) and compressing it, thus exciting the vibrational mode both with the three-color and the two-color mechanism. In this third scenario, we show the results for different compressions of the Stokes beam, demonstrating that a shorter Stokes pulse duration leads to stronger and broader signal for the three-color mechanism. (d) CARS spectrum of toluene (blue curve) and NRB on quartz (orange curve) acquired with 1 ms exposure time. Time-delayed CARS spectrum (blue curve) on toluene obtained changing the delay between the pump and Stokes trains of pulses. SR spectrum of toluene (purple) acquired in 25 s and phase-retrieved spectrum of toluene (light-blue curve). (e, f) The Jablonski diagrams illustrate the two-color (e) and three-color (f) CARS mechanisms.

Our BCARS microscope (see Figure 1a) starts with an amplified ytterbium-fiber laser (Coherent Monaco) generating 270 fs pulses at a 1035 nm wavelength and a 2 MHz repetition rate. With respect to our previous configuration,^29^ we generate the pump pulses with an optimized etalon featuring a narrower Lorentzian transmission spectrum (see Figure S1 in the Supporting Information), thus improving the spectral resolution to <9 cm^–1^ FWHM, corresponding to a 3.7 ps pulse duration (green curve in Figure 1b). The broadband Stokes beam is obtained via WLC generation, focusing the fundamental laser beam with a 75 mm lens in a 10 mm-thick YAG crystal. After the suppression of the residual 1035 nm beam with a long-pass filter (FELH1100, Thorlabs), the Stokes pulses (red-shaded area in Figure 1b) are compressed to ≈20 fs duration by an SF_11_ prism-pair compressor that compensates for the positive group-delay dispersion introduced by the optical elements on the Stokes beam path, including the first microscope objective. We also inserted a mask after the second prism of the compressor to finely tune the extension of the Stokes spectrum, cutting its short-wavelength side, thus limiting the two-color CARS mechanism to the high-frequency region of the vibrational spectrum, as discussed below (see Figure 2).

A delay line mounted on the pump beam path is manually adjusted to temporally overlap the two trains of pulses at the sample plane. A telescope reduces the spot size of the pump beam to match the back aperture of the first microscope objective, while the spot size of the Stokes beam is adjusted by moving the 75 mm achromatic doublet placed right after the YAG crystal for WLC generation. The two beams are then combined with a dichroic mirror and sent to a homebuilt vertical microscope, designed in upright configuration. The sample is raster scanned by an XY motorized translation stage (U-780.DNS, Physik Instrumente), while its z position is controlled with a second motorized XYZ stage (P-545.3R8S, Physik Instrumente), mounted on top of the scanning stage. Sample illumination and light collection are performed by a pair of 0.85-NA air objectives (LCPLN100XIR, Olympus). After the microscope, a short-pass filter (FESH1000, Thorlabs) selects the blue-shifted region of the spectrum that is focused with a 30 mm lens into a spectrometer, consisting of a monochromator (Acton SP2150, Princeton Instruments) equipped with a 600 g/mm grating and a back-illuminated charge-coupled device camera with a 100 × 1340-pixel sensor, featuring high sensitivity in the near-infrared region and a reduced fringe/etalon effect (BLAZE 100HR, Princeton Instruments). Moderate average powers of the Stokes (<10 mW) and pump (<25 mW) beam have been used for all the experiments.

### Control of Two- and Three-Color CARS Mechanisms

In multiplex CARS, the nonlinear signal can be generated by two different mechanisms.^10,31^ In the so-called “two-color” configuration, the vibrational coherence is generated by one interaction with the narrowband pump pulse and one interaction with the broadband Stokes and is then read by a further interaction with the narrowband pump, which generates the anti-Stokes signal. On the other hand, in the “three-color” mechanism, the vibrational coherence is directly generated by the broadband Stokes pulse, according to the ISRS mechanism,^20^ whereby different frequency components of a single broadband pulse simultaneously excite virtual levels and stimulate the emission down to vibrational levels of the ground state. The ISRS process creates a vibrational coherence in all modes whose oscillation period is comparable or longer than the duration of the excitation laser pulse. In turn, this translates into requiring that the bandwidth of the excitation laser is sufficiently large, and the spectral dispersion is minimized so that all its frequencies interact nearly simultaneously with the sample. Once the vibrational coherence has been generated, it can be read out by an interaction with the narrowband pump pulse. The excitation profile in the three-color CARS mechanism depends on the number of permutations of each frequency shift within the Stokes bandwidth and hence vanishes with increasing wavenumber. For this reason, the three-color mechanism emphasizes the lower frequencies and is thus especially useful in the fingerprint region (400–1800 cm^–1^).

These two excitation methods often act in parallel, thus enabling one to collect signals in two different spectral regions simultaneously. To understand their differences, we start from the expression of the CARS signal. The CARS intensity scales quadratically with the third-order nonlinear polarization *P*^(3)^(ω_aS_), generated in the scrutinized sample because of the interaction with the pump (*E*_p_(ω)) and Stokes (*E*_S_(ω)) electric fields. Considering the nonlinear third-order susceptibility of the investigated specimen as χ^(3)^ = χ_R_^(3)^ + χ_NR_^(3)^, where χ_R_^(3)^ and χ_NR_^(3)^ are the resonant and nonresonant term, respectively, the CARS intensity can be expressed as follows:^34^
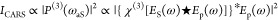
4where ★represents a cross-correlation operation and * stands for the convolution operation. In this equation, the cross-correlation term is responsible for the excitation of vibrational modes whose frequencies match the difference between pump and Stokes frequencies, while the convolution operation with the narrowband pump field, assuming that the cross-correlation is broad enough, defines the spectral resolution of our system. Equation 1 describes the two-color CARS mechanism (Figure 2e). Nevertheless, if the broadband Stokes beam is short enough (shorter than the vibrational oscillation period), an intra-pulse ISRS or three-color excitation mechanism occurs at the sample plane (Figure 2f). After the excitation, the narrowband pump beam acts as a probe, thus determining the final emission at the anti-Stokes frequencies. In the three-color mechanism, eq 1 is modified to
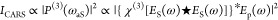
5

In light of the previous discussion, we designed our BCARS experimental setup combining both signal generation mechanisms (Figure 2). We started from a BCARS configuration in which the narrowband pump beam is coupled with the uncompressed broadband Stokes beam (Figure 2b) with bandwidth spanning from 1100 to 1600 nm. In this configuration, the system can acquire spectra covering the whole Raman-active region exploiting only the two-color CARS mechanism. This is demonstrated by the first trace in Figure 2a, which reports a CARS spectrum acquired on toluene sandwiched between two quartz coverslips with a 1 ms exposure time. However, as one can see from the spectrum, the system does not allow us to excite the lower-energy vibrational modes (<600 cm^–1^). To probe also these modes, we carefully tuned the duration of the broadband Stokes pulse and its spectral coverage. We adjusted the pulse duration at the sample plane, modifying the insertion of the second prism so as to maximize the bandwidth (≈400–1400 cm^–1^) and the intensity of the three-color mechanism in the low-frequency region of the spectrum. Then, we also inserted a mask in the plane of the prism compressor where all the colors travel in parallel to carefully adjust the spectral bandwidth, so that the two-color mechanism starts precisely where the three-color mechanism vanishes (≈1400 cm^–1^), to avoid fluctuations on the spectrum due to interference between the two processes.

Using an uncompressed masked Stokes beam with bandwidth ≈1200–1600 nm (Figure 2b), we can obtain spectra for toluene in the same configuration described above, covering the Raman region from 1400 to 3200 cm^–1^ (Figure 2a.2). By compressing the masked Stokes beam, keeping all the other parameters fixed (bandwidth and average power), we obtain different CARS excitation profiles (Figure 2a.3) in the region below 1400 cm^–1^, while the portion above this threshold does not present substantial differences, apart from some intensity variations due to unavoidable slight misalignment of the pump and Stokes beams during the compression of the supercontinuum. The optimum is achieved when the Stokes pulse duration reaches the minimum. This condition results in a spectrum that has the highest intensity and the broadest extension below 1400 cm^–1^. The Stokes pulse duration can be estimated by looking at the highest Raman mode excited through three-color CARS. For the case of toluene, the highest mode is the one at ≈1380 cm^–1^ corresponding to a ≈ 24 fs oscillation period, implying that sub-20 fs Stokes pulses should impinge on the sample, very close to the ≈16 fs TL duration of the pulse.

It is important to notice that the spectral coverage of the two-color mechanism strictly depends on the spectral extension of the Stokes beam that, in our case, reaches ≈1600 nm so that, considering the inter-pulse excitation between pump and Stokes beam, we can visualize Raman modes up to ≈3400 cm^–1^ frequency. However, the spectrometer, due to the use of a 600 g/mm grating, limits the accessible bandwidth to ≈3200 cm^–1^. A 300 g/mm grating would have allowed us to cover a broader spectral range but would have resulted in a lower number of spectral points in the main Raman active region of biological samples (400–3100 cm^–1^).

Once the optimum parameters for the spectral width and the precompression of the Stokes pulses have been set, we can remove the NRB from the spectra via the Kramers–Kronig algorithm and compare the phase-retrieved spectrum (light blue curve) with the SR one (purple curve in Figure 2d). Our results demonstrate that our system enables us to probe both the fingerprint and the CH stretching region of the Raman spectrum. Moreover, we can also perform time-delayed CARS^50^ (blue curve in Figure 2d), by changing the arrival time of the narrowband beam at the sample plane, isolating all the modes excited only via the three-color mechanisms and optically suppressing the NRB without the need of any post-processing algorithm. Moreover, we can also perform time-delayed CARS^50^ (blue curve in Figure 2d), by changing the arrival time of the narrowband pulse at the sample plane, isolating all the modes excited only via the three-color mechanism and optically suppressing the NRB without the need of any post-processing algorithm. The separation between the resonant and nonresonant signals is possible because of their different origin. The resonant contribution in CARS is characterized by a coherence time in the order of picoseconds, since vibrational levels are populated. On the other hand, the NRB arises from electronic contributions, in which only virtual levels are populated. It implies that the NRB is extremely short-lived, mainly contributing for a duration as long as the excitation pulse. By changing the delay between pump and Stokes pulses, the signal generated via two-color CARS vanishes, since both pump and Stokes photons are needed simultaneously at the sample to excite the modes via the two-color mechanism. On the other hand, in three-color CARS, increasing the delay between Stokes and pump, in which the latter acts only as a probe, it is possible to isolate the resonant contribution, featuring a longer coherence time. However, time-delayed CARS also implies a reduction of the resonant signal because of the dephasing of the coherence experienced increasing the time delay, calling for a longer integration time to acquire high signal-to-noise ratio spectra. For these reasons, while time-delayed CARS is particularly beneficial for spectroscopic applications on samples featuring high Raman signals such as solvents, it is not well suited to microscopy on biological samples, since one aims at shorter pixel dwell times to image large fields of view and not damage the biological samples. Therefore, in the following, we will not take advantage of the time-delayed CARS scheme.

### Imaging of HepG2 Cancer Cells

**Figure 3:**
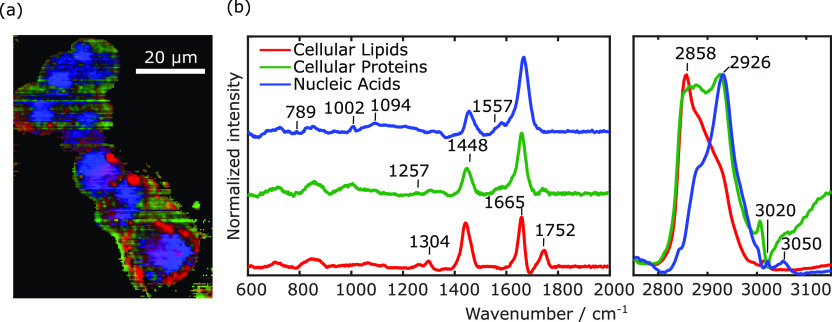
(a) BCARS image of HepG2 cancer cells. The false-color images highlight three different species in the cells. (b) Retrieved endmembers associated with the three species in (a). Imaging settings: 1 ms pixel exposure time; 500 nm pixel size.

We employed the BCARS system to record images of cells, identifying the main sub-cellular components in a label-free manner. The system enabled us to acquire images on HepG2 cells, a well-known human HCC cell line used as a case study of cells reporting specific morphological features (e.g., lipid droplets formation), which usually require a specific fluorescent dye to be visualized. BCARS images were collected at 500 nm pixel size and 1 ms pixel dwell time, collecting the whole Raman spectrum for each pixel. The results presented in Figure 3a demonstrate the capability of our BCARS microscope to map the distribution of lipids (red), proteins (green), and nucleic acids (blue) within the cells. Spectral unmixing methods based on the N-FINDR algorithm allowed us to associate a characteristic spectrum (endmember) to each species (Figure 3b). The endmembers show the typical Raman peaks of cells. In particular, in the fingerprint region, the peak at 789 cm^–1^ is characteristic of DNA, the one at 1002 cm^–1^ is associated with phenylalanine, the band at 1094 cm^–1^ is typical of DNA, the bands between 1200 and 1300 cm^–1^ represent amide III (proteins), the peak at 1304 cm^–1^ is due to CH_2_ twisting (lipids), the peak at 1448 cm^–1^ is associated with CH_2_ bonds, the peak around 1557 cm^–1^ is characteristic of amide II (proteins), the peak at 1665 cm^–1^ is typical of amide I (proteins), and the peak at 1752 cm^–1^ is typical of lipids (ester vibration). In the C–H stretching region, the spectra feature broader features. The peak around 2858 cm^–1^ (see red line) is related to the CH_2_ symmetric stretch of lipids; the peak at 2926 cm^–1^ (see green line) is related to the symmetric CH_3_ stretch, primarily in proteins; and finally, the peak at 3020 cm^–1^ (see red line) is related to the unsaturated =CH stretch of lipids. These results demonstrate that BCARS microscopy is able to image and detect the accumulation of lipids occurring in HepG2 cells and differentiate the different subcellular components, including nuclei, cytoplasm, and lipid droplets.

### Tissue Imaging

We extended the use of the BCARS system to image the liver from mice with NASH and compare it to control liver. NASH is a nonalcoholic fatty liver disease affecting 8–10% of Europeans. A decrease in cell autophagic activity has been linked to the initial development of steatosis and its progression to NASH and HCC.^51^ NASH is characterized by (i) ballooned hepatocytes, (ii) fatty hepatocyte foci, (iii) inflammation, and (iv) increased hepatocyte cell death. The pathogenesis of NASH for the optimization of prevention and treatments is usually studied in mice using in vivo and ex vivo complex and potentially disruptive approaches, and it requires enzymological, biochemical, histological, transcriptomic, and omics analyses of the liver. In addition, the study of the subcellular distribution and composition of lipids in NASH is challenging and usually requires specific fluorescent labelling or the use of destructive approaches such as mass spectroscopy. Our BCARS microscope allows retrieving label-free and multidimensional information on the spatial distribution of these compartments and their biochemical composition.

**Figure 4:**
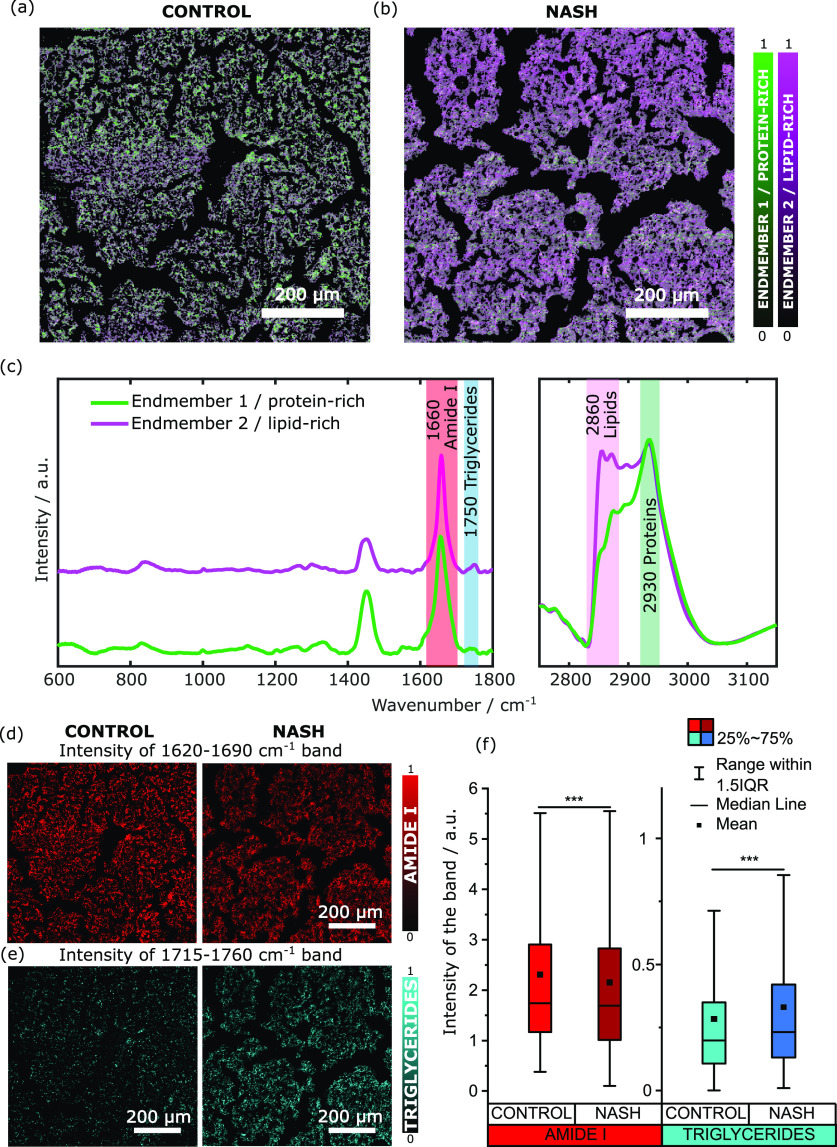
(a, b) 800 × 800 μm^2^ false-color BCARS images on control (a) and NASH (b) liver slices obtained through N-FINDR analysis on the merged data after noise and NRB removal. (c) Spectra (endmembers) associated with the species differentiated in (a, b). (d, e) Concentration maps from the data after the denoising and NRB removal obtained integrating the band 1620–1690 cm^–1^ of amide I (d) and the band 1715–1760 cm^–1^ of triglycerides (e). (f) Box plot reporting the intensity of the band of amide I, integrated in the 1620–1690 cm^–1^ region, and of the band of triglycerides, integrated in the 1715–1760 cm^–1^ region. Data are shown as boxes and whiskers. Each box represents the 25th to 75th percentiles [interquartile range (IQR)]. Dots inside the box are the mean; lines inside the boxes represent the median. The whiskers represent the lowest and highest values within the boxes ±1.5× the IQR. *** refers to *P*-value <0.001 resulting from a Mann–Whitney test with null hypothesis of equal median between the two samples. Imaging settings: 3 ms pixel exposure time, 2 μm pixel size.

For this purpose, we imaged a large field of view of 10 μm-thick liver tissue slices taken from a control sample, obtained with RCD diet, and from a liver isolated from a NASH mouse model, obtained with MCD diet (detailed in the Sample Preparation part of Materials and Methods section). In both samples, we imaged a 800 × 800 μm^2^ region and set a pixel size of 2 × 2 μm^2^ with a 3 ms pixel dwell time (see Figure 4). The hyperspectral images of the wide scanned areas have been analyzed following the same procedure described above. After denoising and NRB removal, we extracted the most abundant spectral features (called endmembers) applying the unsupervised N-FINDR algorithm to both images analyzed at once as a single hyperspectral data cube, in order to describe the two datasets using the same spectra. N-FINDR clustering identified two main endmembers (see Figure 4c) reporting protein-rich and lipid-rich signatures in both tissues. The associated abundances in each dataset are plotted in Figure 4a,b. In the spectra, we can identify the main peaks in the fingerprint region at 831 cm^–1^ (tyrosine), 1002 cm^–1^ (phenylalanine), 1125 cm^–1^ (CC stretch of proteins and lipids), 1257 cm^–1^ (lipids band), 1325 cm^–1^ (lipids), 1450 cm^–1^ (CH_2_ bending), 1567 cm^–1^ (amide II), 1660 cm^–1^ (amide I), and 1750 cm^–1^ (ester vibration). In the high-wavenumber regions, the main peaks are the one at 2850 cm^–1^ associated with the CH_2_ symmetric stretch of lipids, at 2874 cm^–1^ typical of the CH_2_ asymmetric stretch of lipids, and at 2935 cm^–1^ of the CH_3_ stretching of proteins. The analysis clearly shows that the chemical composition of the two tissue slices differs in the lipid-rich component, represented by endmember 2 (purple curve in Figure 4c). Indeed, the NASH sample (Figure 4b) presents a higher concentration of lipids with respect to the control samples, as qualitatively shown by the predominant purple areas. Contrarily, in the control sample, the dominant component is the protein-rich one, represented by endmember 1 (green curve in Figure 4c).

The clustering analysis based on the N-FINDR algorithm allowed us to unveil the enrichment of lipids in the NASH model. In particular, endmember 2 contains a specific band centered at 1750 cm^–1^, corresponding to the ester vibrations typical of triglycerides, while both endmembers feature the big peak of amide I at 1660 cm^–1^. To better investigate the presence and distribution of amide I and the lipid class of triglycerides in the two liver samples, we integrated the associated bands in the fingerprint region and we produced the relative concentration maps (Figure 4d,e). The two images clearly show an accumulation of triglycerides in the NASH tissue with respect to the case of the control sample, which in turn features a slightly higher concentration of amide I. This is more evident if we group the pixel intensities in a box-plot graph (Figure 4f) after removing for both images the pixels of the substrate where no tissue is present and removing the outliers, setting the same threshold to not distort the statistical analysis. This semi-quantitative analysis demonstrates that the control tissue has a higher median and mean value in the band of amide I, while the NASH tissue features higher median and mean value in the band of triglycerides. This is confirmed by the Mann–Whitney tests that showed *P* < 0.001. These results are consistent with previous studies^52−55^ that report an accumulation of triglycerides in nonalcoholic fatty liver disease and NASH and pave the way for an exploration of the role of triglycerides in different NASH phenotypes using BCARS microscopy.

Finally, we demonstrated that our technique can be used to identify the tumor regions and differentiate them from the healthy portions in thin tissue slides.^1,33,56−58^ Nowadays, the identification of cancer and the corresponding changes in the tissue morphology is based on staining techniques. The most common one is H&E, which employs the dyes hematoxylin (marking the nuclei in purple/blue) and eosin (providing a pink color to the cytoplasm and the extracellular connective tissue matrix). Using H&E staining, pathologists recognize morphological changes, patterns, shapes, and cellular structures. However, H&E staining is labor- and time-intensive and does not provide chemical specificity. Instead, BCARS enabled us to avoid the necessity of staining samples and to transcend the purely morphological aspects by retrieving information on chemical composition of distinct cellular compartments.

**Figure 5:**
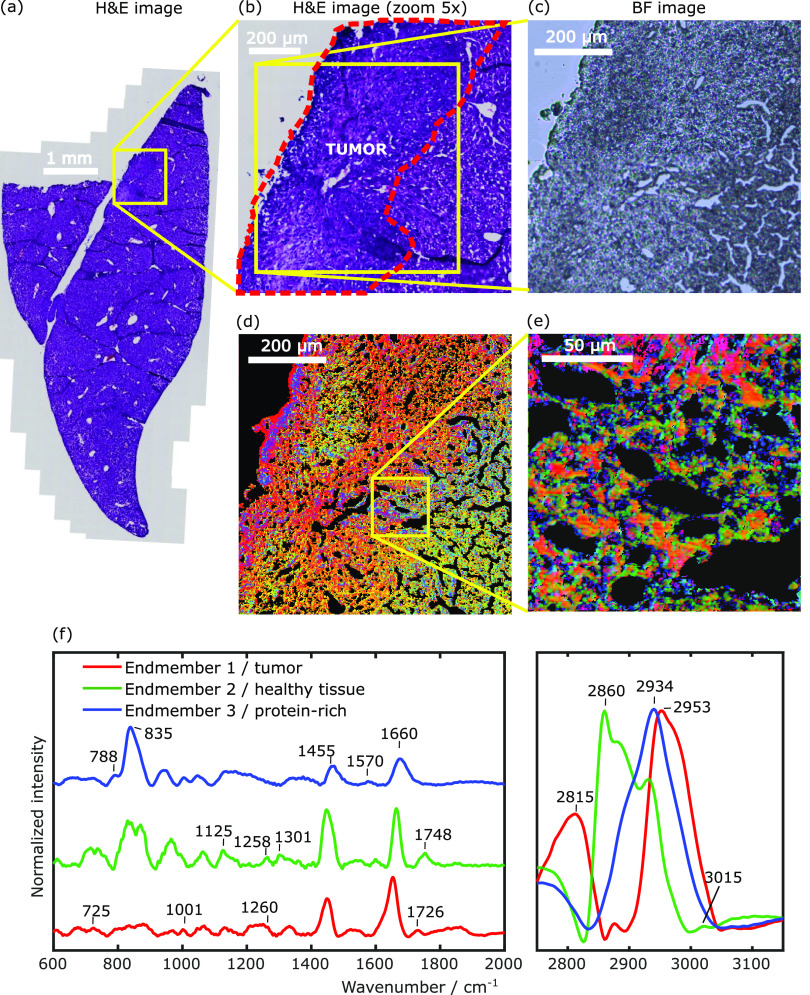
BCARS imaging on tumor liver sample. (a) H&E image of the investigated slice. (b) 5× Zoom on the region highlighted in yellow. (c) Bright-field image of the scanned region. (d, e) 800 × 800 μm^2^ (d) and 150 × 150 μm^2^ (e) BCARS images on tumor liver slice obtained through spectral unmixing analysis after noise and NRB removal, with 3 ms pixel exposure time and 2 μm (d) and 500 nm (e) pixel sizes, respectively. (f) Retrieved endmembers associated with the species in (d) and (e).

To assess the system capabilities in tumor recognition, we imaged liver slices of a orthotopic mouse model of HCC. This model involved the direct intrahepatic injection of HCC tumor cells into the liver, i.e., their natural habitat. We cut two adjacent 10 μm-thick slices: the first one was treated with H&E staining and used for standard tumor assessment (see Figure 5a,b), while the second was directly fixed onto a quartz coverslip without any treatment, sandwiched with a second quartz coverslip, and imaged with our microscope. Before the BCARS experiments, we acquired a brightfield image of the unstained slice to visualize its morphological details (see Figure 5c), which resemble (although with slight differences) those of the H&E-stained adjacent slide. We then recorded BCARS images over an 800 × 800 μm^2^ field of view with 2 μm pixel size and 3 ms pixel dwell time. After data post-processing, the N-FINDR algorithm was used to produce the BCARS false-color images (Figure 5d) by overlapping the concentration maps associated with three main spectral features (endmembers, Figure 5f) identified in the sample. The BCARS image was then correlated with the annotation performed by pathologists on the H&E-stained slice (Figure 5a,b). The first component (red) mainly identifies the tumor; the second component (green) mainly colocalizes with healthy liver tissue surrounding the tumor; the third component (blue) is mainly localized at the interface between tumor and healthy liver but also in correspondence with the outer layer of the tissue slice, mainly represented by connective tissue. The endmember associated with the healthy region (green, Figure 5f) features relatively high lipid signals (peaks at 1301, 1455, 1748 cm^–1^, and especially the prominent CH_2_ band at 2860 cm^–1^) but also spectral features of connective tissue proteins, like collagen and elastin (mainly due to proline and hydroxyproline peaks, in the 810–840 cm^–1^ region and around 1258 cm^–1^).^59,60^ The endmember associated with the tumor (red, Figure 5f) shows spectral features that differ from those detected in the noncancerous tissue, presenting predominant protein-related peaks, at 1660 and around 2950 cm^–1^, and lower signals related to lipids and connective tissue. The increase of protein-rich molecules (and the decrease of lipid-rich molecules) in the tumor region of the liver sample is in accordance with the accumulation of tumor cells typically invading the lipid-rich matrix of the liver, thus increasing the number of undifferentiated tumor cells mainly characterized by a high nuclear:cytoplasmic ratio and scarce production of lipids at both intracellular and extracellular levels. The third endmember (blue, Figure 5f) is again characterized by spectral features mainly associated with proteins (peaks at 1450 and 1660 cm^–1^ and predominant peak at 2934 cm^–1^) including those associated with the connective tissue (broad peak centered around 810–840 cm^–1^). Considering that collagen is the main component of connective tissue, we performed a second harmonic generation (SHG) image of the same tissue slice utilized for BCARS analysis, aiming to verify the colocalization of fibrillar collagen with the three regions detected using BCARS (see Figure S2 in the Supporting Information). Our data indicate that collagen is highly expressed in the middle-left portion of the image under investigation, in correspondence with the tumor, in agreement with evidence reporting the increase of collagen induced by cancer.^61^ At the same time, the tumor-related endmember (red) identified by BCARS does not show intense collagen-like features (Figure 5f), which, in turn, are more evident in the green and blue endmembers. This is consistent with the fact that collagen fibrils are extracellular components locally surrounding the tumor cells, and fundamental components of the tumor microenvironment. Moreover, this is also confirmed by the high-resolution BCARS Raman image reported in Figure 5e, showing that cellular bodies of tumor cells (red) are surrounded by a complex microenvironment and extracellular network (green and blue features). What distinguishes the blue and the green endmembers is a different balance between proteins and lipids, as clearly visible in the high-wavenumber region: the green regions contain more lipid-rich components, and the blue regions contain more protein-rich components. This difference can be associated with different sub-components of the tissue microenvironment, including cells, extracellular matrix, and lipids. Further studies specifically dedicated to the investigation of the tumor microenvironment by BCARS imaging will be necessary to better describe its composition. These preliminary data show that the extended spectral range of BCARS not only allows the recognition of the tumor-like biochemical features without specific labelling but also permits the characterization of the tumor microenvironment, with potential implications in the research and clinical fields.

## Conclusions

In this work, we reported a new experimental approach to multiplex CARS based on a 2 MHz repetition rate-amplified femtosecond ytterbium laser system used to generate white-light supercontinuum in bulk media. By pre-compressing the generated broadband Stokes pulses in the infrared, we could obtain at the sample plane sub-20 fs pulses that, coupled with the narrowband 3.7 ps pump pulses, generate a CARS signal spanning the entire Raman spectrum of biological samples. The broadband anti-Stokes signal is generated by two nonlinear processes that act in parallel, namely, the three-color and the two-color mechanisms. The former derives from the intra-pulse interaction of the Stokes pulses and generates an excitation profile, which depends on the number of permutations of the colors inside the Stokes bandwidth. We exploited this mechanism to cover the lower frequency portion of the spectrum (500–1400 cm^–1^). The latter is the result of the inter-pulse interaction between the pump and the Stokes beam and generates an excitation profile as broad as the Stokes pulse spectrum. We employed this mechanism to cover the higher-frequency portion of the Raman spectrum (1400–3100 cm^–1^).

Thanks to the new optical design, the system enables us to acquire damage-free images of cells and tissues with millisecond pixel dwell time, delivering highly informative chemical maps. We have shown images on HepG2 cancer cells acquired with 1 ms exposure time, distinguishing lipids, proteins, and nuclei and highlighting the main peaks in each of their spectra. Then, we broadened the application of the technique imaging control and NASH liver tissue slices, providing chemical images over very large fields of view (800 × 800 μm^2^) that we qualitatively and semi-quantitatively compared.

Eventually, we demonstrated the potential of the system to be used in histopathological settings, imaging a mouse liver model implanted with HCC. Comparing the results with the H&E image of an adjacent slice, we confirm that our system not only is capable of localizing the tumor and providing morphological details on the sample but also offers precious information on the chemical composition of each region of the sample. Moreover, we demonstrated that at the excitation power levels that we employ, raster scanning of the same field of view did not damage the investigated sample, preserving the chemical information and confirming that our technique is nondestructive.

These results open the path toward the use of full-spectrum CARS microscopy for studying the origin of diseases at both the cellular and tissue levels and for diagnostic purposes. The possibility of accessing the vibrational modes of the fingerprint region allows one to conduct accurate chemical analysis targeting specific chemical species that cannot be isolated from the broad peaks of the CH-stretching region, as we have shown with the triglycerides in the NASH sample. Compared to standard techniques that employ labels, such as histochemistry and fluorescence microscopy, our system does not require any sample preparation nor the use of markers that often alter the chemical structure or undergo photobleaching, preventing repeated imaging of the same region of interest. Compared to other label-free techniques, such as SR, BCARS provides much higher acquisition speed and does not suffer from sample autofluorescence. Eventually, we envisage that the acquisition speed of our BCARS apparatus, thanks to the high energies of the pump and Stokes pulses, can be further improved by employing either line scanning or wide-field illumination.

## Data Availability

Data underlying the results presented in this paper are available in Zenodo; see ref (62).
