# Supplementary material for: Full-Spectrum CARS Microscopy of Cells and Tissues with Ultrashort White-Light Continuum Pulses
Source: J Phys Chem B. 2023 May 17;127(21):4733–45. doi: 10.1021/acs.jpcb.3c01443 (PMC10240501; doi:10.1021/acs.jpcb.3c01443)
Supplement: Supplementary file 1 — jp3c01443_si_001.pdf [file jp3c01443_si_001.pdf]

Full-spectrum CARS Microscopy Of Cells And Tissues With Ultrashort White-light Continuum Pulses

Federico Vernuccio,[1] Renzo Vanna, [2] Chiara Ceconello, [1] Arianna Bresci ,[1] Francesco Manetti, [1] Salvatore Sorrentino,[1] Silvia Ghislanzoni,[3] Flavia Lambertucci,[4,5] Omar Motiño,[4,5] Isabelle Martins,[4,5] Guido Kroemer,[4,5,6] Italia Bongarzone,[3] Giulio Cerullo,[1,2] and Dario Polli [1,2,]*

[1]Department of Physics, Politecnico di Milano, P.zza Leonardo da Vinci 32, 20133 Milan, Italy

[2]CNR-Institute for Photonics and Nanotechnologies (IFN-CNR), P.zza Leonardo Da Vinci 32, 20133 Milan, Italy

[3]MALDI-imaging Lab, Department of Advanced Diagnostics, Fondazione IRCCS Istituto Nazionale dei Tumori, Via G. Amadeo 42, 20133, Milan Italy.

[4]Centre de Recherche des Cordeliers, Equipe labellisée par la Ligue contre le cancer, Inserm U1138, Université Paris Cité, Sorbonne Université, 75006 Paris, France.

[5]Metabolomics and Cell Biology Platforms, Gustave Roussy, 94805 Villejuif, France.

[6]Institut du Cancer Paris CARPEM, Department of Biology, Hôpital Européen Georges Pompidou, France-HP, 75015 Paris, France.

Email: dario.polli@polimi.it

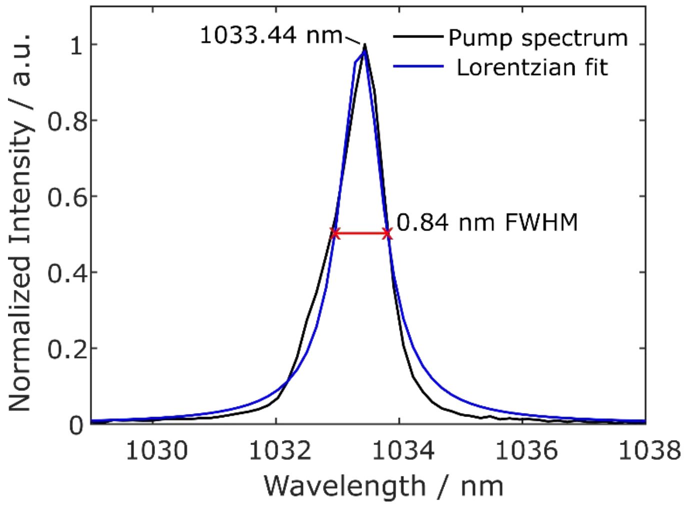

Figure S1 Pump spectrum (black line) after the etalon fitted with a Lorentzian function (blue line) centered at 1033.4 nm and with 0.84 nm full width at half maximum (FWHM). The mean absolute percentage error between the measured spectrum and the Lorentzian fit is 2,11 %.

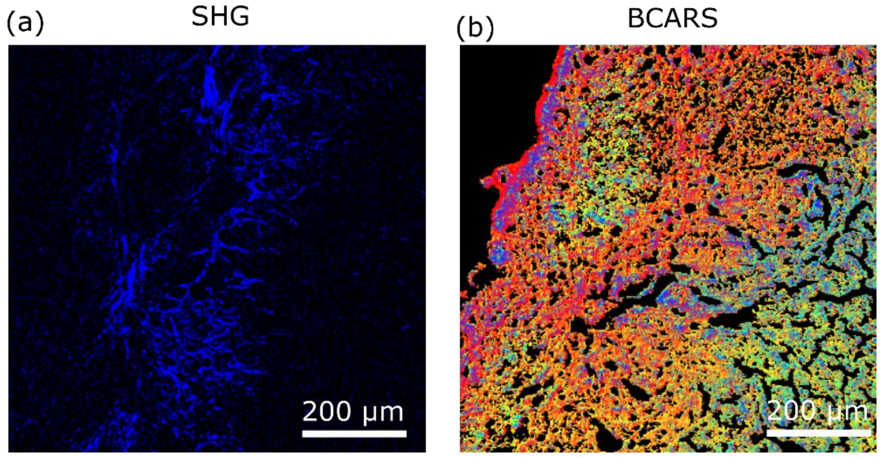

Figure S2 SHG image (a) and BCARS image (b) on tumor liver sample. Image settings: 800 x 800 µm^2, pixel dwell time: 15 ms/pixel for SHG detected with the spectrometer, 3 ms/pixel for BCARS.
